# Supplementary material for: Geographic and Socioeconomic Influence on Knowledge and Practices Related to Antimicrobial Resistance among Smallholder Pig Farmers in Uganda
Source: Antibiotics (Basel). 2022 Feb 15;11(2):251. doi: 10.3390/antibiotics11020251 (PMC8868422; doi:10.3390/antibiotics11020251)
Supplement: Supplementary file 1 [file antibiotics-11-00251-s001.zip › antibiotics-1581440-supplementary-file.pdf]

antibiotics

Background information, Supplementary material

Demographics and Socioeconomic Characteristics

The demographic and socioeconomic data of the investigated farms are presented in Table S1. There was no difference in gender distribution among the respondents between the two districts, with both districts having a higher proportion of females than males. The mean age of the respondents was around 40 years in both districts, but higher in Mukono than in Lira ($p = 0.000$). In Lira the education level of the respondent was generally lower, with a larger proportion that had never went to school ($p = 0.025$) and a lower proportion that had gone through secondary school ($p = 0.0029$).

Almost all respondents in both Lira and Mukono were engaged in the management of the livestock at the farm. In Mukono, however, larger proportions of the respondents were also the farm owner ($p = 0.000$) and/or involved with marketing ($p = 0.000$) compared to Lira. In Lira, male household heads had the main responsibility for the pigs to a larger extent compared to Mukono ($p = 0.030$), where the opposite was true for female household heads ($p = 0.011$). Even though not a majority of farms in either district, having hired workers was more common in Mukono than in Lira ($p = 0.043$).

In neither district was pig keeping the main income source; instead, it was crop farming, though more common in Lira than in Mukono ($p = 0.000$). To be self-employed outside the farm or to have a salaried employment were both more common in Mukono than in Lira ($p = 0.027$ and $p = 0.0019$). In Lira, more than half of the respondents answered that livestock contributed to half or more of the household's income compared to only one third in Mukono ($p = 0.000$).

Table S1. Comparison of demographics and socioeconomic characteristics on smallholder pig farms in Lira and Mukono districts, Uganda.

Variable	Category	Lira	Mukono	*p*-value
		% (*n*)	% (*n*)	
Sex of the respondent[1]	Male	41.9 (96)	43.2 (101)	0.777
	Female	58.1 (133)	56.8 (133)	0.777
Age of the respondent (years)[1]	Mean	38.7	45.3	0.000
Education level of the respondent[1]	Never went to school	11.4 (26)	5.6 (13)	0.025
	Primary education	57.6 (132)	41.0 (96)	0.00035
	Secondary school	23.6 (54)	36.3 (85)	0.0029
	Vocational training	4.8 (11)	8.6 (20)	0.103
	University	2.6 (6)	8.6 (20)	0.0051
Role of the respondent in relation to livestock (multiple choice)[1]	Management	96.5 (221)	92.7 (217)	0.071
	Marketing	44.5 (102)	80.3 (188)	0.000
	Owner	17.9 (41)	88.0 (206)	0.000
Person with the main responsibility for the pigs[2]	Household head (man)	41.1 (94)	31.3 (73)	0.030
	Household head (woman)	31.9 (73)	43.4 (101)	0.011
	Joint responsibility (couple)	24.0 (55)	20.2 (47)	0.319
	Daughter	0.4 (1)	2.2 (5)	n/a
	Son	1.8 (4)	2.6 (6)	n/a
	Employee	0.4 (1)	0.4 (1)	n/a

	Other relative	0.4 (1)	0 (0)	n/a
Hired workers on the farm[1]	Yes	15.3 (35)	22.6 (53)	0.043
	No, family members only	84.7 (194)	77.4 (181)	0.043
Main source of income for the household[3]	Pig keeping	10.1 (23)	9.9 (23)	0.943
	Crop farming	57.5 (131)	32.2 (75)	0.000
	Cattle keeping	3.1 (7)	6.9 (16)	0.062
	Small ruminant keeping	0.9 (2)	0 (0)	n/a
	Poultry keeping	0 (0)	1.7 (4)	n/a
	Salaried employment	7.0 (16)	16.3 (38)	0.0019
	Self-employed off farm	15.8 (36)	24.0 (56)	0.027
	Casual labouring	3.1 (7)	2.6 (6)	n/a
	Boda Boda	1.3 (3)	2.2 (5)	n/a
	Other	1.3 (3)	4.3 (10)	n/a
Livestock contributes to[1]	Half or more of the household income	54.2 (124)	35.9 (84)	0.0001
	Less than half of the household income	42.4 (97)	61.1 (143)	0.0001
	Does not contribute to the household income	3.5 (8)	3.0 (7)	n/a

[1] Lira *n* = 229 Mukono *n* = 234, [2] Lira *n* = 229 Mukono *n* = 233, [3] Lira *n* = 228 Mukono *n* = 233. n/a = not applicable, comparative statistic analysis could not be performed

Farm Characteristics

The data on farm characteristics of the investigated farms are presented in Table S2. Both the median and mean numbers of pigs per farm were lower in Lira than in Mukono ($p = 0.000$). In Lira, it was generally more common than in Mukono to, besides pigs, keep cattle ($p = 0.000$), small ruminants ($p = 0.0018$) and poultry ($p = 0.015$). However, a majority of the farms in both districts kept more than one species, even though the proportion was higher in Lira ($p = 0.0014$).

It was more common in Lira to keep the pigs tethered compared to Mukono ($p = 0.000$), while keeping the pigs housed was more common in Mukono than in Lira ($p = 0.000$). Keeping the pigs free-range was non-existent at the farms in Mukono, but was used in 16 farms (7%) in Lira. More than half of the farms in both districts sold live pigs, but the proportion was higher in Mukono ($p = 0.000$).

Table S2. Comparison of farm characteristics in smallholder pig farms in Lira ($n = 229$) and Mukono ($n = 234$) districts, Uganda.

Item	Category	Lira	Mukono	*p*-value
Number of pigs per farm	Mean	3.3	6.5	0.000
	Median	2	4	n/a
	Range	1-35	1-55	n/a
		**% (*n*)**	**% (*n*)**	
What livestock do you have (other than pigs)? (multiple choice)	Cattle	54.6 (125)	35.5 (83)	0.000

	Small ruminants	59.0 (135)	44.4 (104)	0.0018
	Poultry	73.4 (168)	62.8 (147)	0.015
Farms with more than one species		88.2 (202)	76.9 (180)	0.0014
Livestock production system (for pigs)	Free range	7.0 (16)	0 (0)	n/a
	Tethered	72.9 (167)	26.9 (63)	0.000
	Housed	20.1 (46)	73.1 (171)	0.000
Farms that sell live pigs		50.2 (115)	87.2 (204)	0.000

n/a = not applicable, comparative statistic analysis could not be performed

Feeding and Manure Management Routines

The data on practices regarding feed and manure at the investigated farms are presented in Table S3. Even though ranging between half to almost all farms in both districts, using grains and/or crop residues, household and/or restaurant waste and feed mixed at the farm was more common in Mukono than Lira (p = 0.000, p = 0.0003 and p = 0.0078, respectively). Pasture and scavenging, on the other hand, was more common in Lira than in Mukono (p = 0.000). Even though less common than other feed products in both districts, the proportion of farms that used commercial feed or pre-mix was larger in Mukono than in Lira (p = 0.0018).

In Lira, the most common ways to manage the pig manure were to discard it into the environment or to leave it in open air. Both these practices were more common in Lira than in Mukono (p = 0.000 and p = 0.000). In Mukono, on the other hand, the majority of farmers used the manure as fertilizer (75%), compared to Lira, where only 19% used the manure for this purpose (p = 0.000).

Table S3. Comparison of feeding and manure management routines in smallholder pig farms in Lira (n = 229) and Mukono (n = 234) districts, Uganda.

Item	Category	Lira	Mukono	*p*-value
		% (*n*)	% (*n*)	
Feed product used for pigs (multiple choice)	Pasture/scavenging	70.3 (161)	19.2 (45)	0.000
	Waste (household/ restaurant etc.)	49.8 (114)	66.2 (155)	0.0003
	Grains/crop residues	81.7 (187)	97.0 (227)	0.000
	Feed mixed at farm	54.6 (125)	68.1 (156)	0.0078
	Commercial/pre-mix	7.9 (18)	17.5 (41)	0.0018
Management of pig manure	Leave on farm (do nothing)	11.4 (26)	9.4 (22)	0.491
	Discard into environment	34.1 (78)	9.0 (21)	0.000
	Open air	34.5 (79)	2.1 (5)	0.000
	Used as fertilizer	19.2 (44)	74.8 (175)	0.000
	Use for fuel (incl. biogas)	0 (0)	1.3 (3)	n/a
	Sold for cash	0 (0)	2.6 (6)	n/a
	Taken by other farmers	0.9 (2)	0.9 (2)	n/a

n/a = not applicable, comparative statistic analysis could not be performed

Disease Occurrence

The data on disease issues and diagnosis of disease at the investigated farms are presented in Table S4. During the 12-month period prior to the study, respiratory and digestion/intestinal disorders were the main disease problems among the pigs at the farms in Lira, and they were more common than in Mukono (p = 0.000 and p = 0.021). Further, sudden death among the pigs was also more common in Lira than in Mukono (p = 0.000). Notably, 52% of the respondents in Mukono reported that they had no disease among their pigs the past 12 months compared to only 19% in Lira (p = 0.000).

The proportion of respondents that had experienced disease among their animals (all species) during the past two weeks was higher in Lira than in Mukono (p = 0.000), and in both districts, it was most commonly the pigs that had been sick.

Table S4. Comparison of self-reported disease issues in smallholder pig farms in Lira and Mukono districts, Uganda.

Item	Category	Lira	Mukono	*p*-value
		% (*n*)	% (*n*)	
Main disease problems in pigs, last 12 months[1]	Respiratory	23.6 (54)	3.0 (7)	0.000
	Digestive/ intestinal	24.9 (57)	16.2 (38)	0.021
	Reproductive	1.3 (3)	3.0 (7)	n/a
	Mastitis	0 (0)	0.4 (1)	n/a
	Sudden death	14.8 (34)	2.6 (6)	0.000
	Skin disease/ Wounds	3.9 (9)	6.8 (16)	0.166
	External parasites	5.2 (12)	2.6 (6)	n/a
	Neurologic signs	2.6 (6)	1.3 (3)	n/a
	No disease	18.8 (43)	51.7 (121)	0.000
	Other	4.8 (11)	12.4 (29)	0.0037
Have any animals been sick the last 2 weeks (all species)?[1]	Yes	55.0 (126)	28.6 (67)	0.000
Which animals have been sick in the last 2 weeks? (multiple choice)[2]	Pigs	44.4 (56)	53.7 (36)	0.219

[1] Lira n = 229 Mukono n = 234, [2] Lira n = 126 Mukono n = 67. n/a = not applicable, comparative statistic analysis could not be performed
